# Dysuria Following Stereotactic Body Radiation Therapy for Prostate Cancer

**DOI:** 10.3389/fonc.2015.00151

**Published:** 2015-07-03

**Authors:** Einsley-Marie Janowski, Thomas P. Kole, Leonard N. Chen, Joy S. Kim, Thomas M. Yung, Brian Timothy Collins, Simeng Suy, John H. Lynch, Anatoly Dritschilo, Sean P. Collins

**Affiliations:** ^1^Department of Radiation Medicine, Georgetown University Hospital, Washington, DC, USA; ^2^Department of Urology, Georgetown University Hospital, Washington, DC, USA

**Keywords:** dysuria, prostate cancer, stereotactic body radiation therapy, AUA, expanded prostate index composite, CyberKnife, quality of life

## Abstract

**Background:**

Dysuria following prostate radiation therapy is a common toxicity that adversely affects patients’ quality of life and may be difficult to manage.

**Methods:**

Two hundred four patients treated with stereotactic body radiation therapy (SBRT) from 2007 to 2010 for localized prostate carcinoma with a minimum follow-up of 3 years were included in this retrospective review of prospectively collected data. All patients were treated to 35–36.25 Gy in five fractions delivered with robotic SBRT with real time fiducial tracking. Dysuria and other lower urinary tract symptoms were assessed via Question 4b (Pain or burning on urination) of the expanded prostate index composite-26 and the American Urological Association (AUA) Symptom Score at baseline and at routine follow-up.

**Results:**

Two hundred four patients (82 low-, 105 intermediate-, and 17 high-risk according to the D’Amico classification) at a median age of 69 years (range 48–91) received SBRT for their localized prostate cancer with a median follow-up of 47 months. Bother associated with dysuria significantly increased from a baseline of 12% to a maximum of 43% at 1 month (*p* < 0.0001). There were two distinct peaks of moderate to severe dysuria bother at 1 month and at 6–12 months, with 9% of patients experiencing a late transient dysuria flare. While a low level of dysuria was seen through the first 2 years of follow-up, it returned to below baseline by 2 years (*p* = 0.91). The median baseline AUA score of 7.5 significantly increased to 11 at 1 month (*p* < 0.0001) and returned to 7 at 3 months (*p* = 0.54). Patients with dysuria had a statistically higher AUA score at baseline and at all follow-ups up to 30 months. Dysuria significantly correlated with dose and AUA score on multivariate analysis. Frequency and strain significantly correlated with dysuria on stepwise multivariate analysis.

**Conclusion:**

The rate and severity of dysuria following SBRT is comparable to patients treated with other radiation modalities.

## Introduction

Over 200,000 men were diagnosed with prostate cancer in the United States in 2014, making prostate cancer the most common cancer in men (1). Localized prostate cancer is typically treated with either surgery or radiation, with external beam radiation therapy (EBRT) and brachytherapy being the most commonly utilized radiation treatment modalities. Selection of treatment modality depends on a number of factors, including age, performance status, risk stratification, and patient preference. As prostate cancer is associated with a high-cure rate and a long natural history, treatment side effects may have a large impact on quality of life (QOL). Indeed, studies have revealed that patient desire for curative therapy can be heavily influenced by treatment-related changes in QOL (2, 3).

Urinary symptoms are a primary determinate of QOL following prostate radiotherapy (4). Dysuria is a clinical problem associated with benign prostatic enlargement (BPH) and/or prostatitis (5). It is a commonly reported toxicity following pelvic radiation therapy and may be difficult to manage (6). Patients with radiation-induced dysuria describe symptoms of burning or pain with urination. The etiology of radiation-induced dysuria is unknown, but may involve inflammation and mucosal loss at the urethra and bladder neck (6). The risk of dysuria appears to be dependent upon a number of factors, including the prostate volume, the volume of the urethra receiving a high-radiation dose, and delayed use of alpha-blockers (7, 8).

Dysuria is often an acute symptom that peaks within the first few months following treatment and resolves with time (4). Accurate capture of the patient reported experience is heavily dependent on the assessed time points, with some reports potentially missing the full extent of dysuria when the first assessment is not within the first weeks to month post-treatment (4, 9). Other factors that may influence the reporting of dysuria include the severity of the symptom, with only the most severe symptomatology being reported, and the questions utilized to capture the data, with only some forms having specific questions related to dysuria.

Despite the complexities of capturing dysuria information and inter-researcher differences in data capture techniques, there does appear to be differences in both the severity and the temporal aspects of the peak and resolution of dysuria dependent upon the radiation technique employed (4, 10). Following conventionally fractionated EBRT, the frequency of moderate to severe dysuria is 12, 5, and 1% at 2, 6, and 12 months post-treatment, respectively (4). In comparison, brachytherapy patients reported moderate to severe dysuria frequency of 24, 11, and 11% at 2, 6, and 12 months, respectively (4). Indeed, dysuria is a commonly reported side effect of brachytherapy treatment (9, 11–13), with frequencies of up to 85–88% at 1 month following treatment (9, 13), decreasing to 50% at 6 months (9). For men who reported dysuria after brachytherapy, the dysuria persisted for 36 months prior to resolution (14). While urethral dose has been shown to be a statistically significant predictor of urinary morbidity (15), studies looking specifically at clinical, treatment, and dosimetric variable predictors of brachytherapy-related dysuria have failed to demonstrate significance (9, 14). Only higher post-implant American Urological Association (AUA) scores significantly predicted for dysuria (14). Merrick et al. showed that prophylactic tamsulosin significantly reduced dysuria rates after brachytherapy (9), and Prosnitz et al. showed that tamsulosin relieved the symptoms of radiation urethritis after EBRT (16).

Radiation dosing and fractionation for the curative treatment of prostate cancer are areas of active clinical investigation. While standard radiation dosing involves daily treatment for 8–9 weeks, stereotactic body radiation therapy (SBRT) allows treatment over a shorter time span, with delivery of fewer, high-dose fractions of radiation. Early data from trials of SBRT for treatment of localized prostate cancer show SBRT to be safe and effective (17–25). However, it is still uncertain whether the use of large fraction sizes could increase the incidence and severity of urinary morbidity, such as dysuria. The goal of this study is to report the incidence and severity of dysuria following SBRT for prostate cancer.

## Materials and Methods

### Patient selection

Eligible patients included those with histologically confirmed prostate cancer without evidence of involved lymph nodes, clinical stage T3 disease, distant metastases, and/or prior pelvic radiation. Quality of life (QOL) data were prospectively collected for all patients per our institutional protocol. This study was performed with full Internal Review Board (IRB) approval.

### SBRT treatment planning and delivery

Our institutional SBRT treatment planning and delivery has been previously described (17, 26). Briefly, several days after placement of three to four gold markers, the patients underwent magnetic resonance (MR) and computed tomography (CT) imaging. The MR and CT images were then fused and used for treatment planning. The prostate and proximal seminal vesicles made up the clinical target volume (CTV); this volume was then expanded 3 mm posteriorly and 5 mm in all directions to define the planning target volume (PTV). Patients were treated with our institutional SBRT monotherapy protocol to 35–36.25 Gy in five fractions of 7–7.25 Gy prescribed to the PTV; the tumor equivalent dose in 2 Gy fractions (EQD2) is 85–90 Gy assuming an alpha/beta ratio of 1.5.

Plans were inhomogeneous by design to minimize dose to adjacent critical structures. Dose–volume histogram (DVH) analysis of critical structures, including the bladder and membranous urethra, was performed using Multiplan (Accuray Inc., Sunnyvale, CA, USA) inverse treatment planning. Treatment DVH goals included a maximum dose of 37 Gy to <5 cc of the bladder and <50% of the membranous urethra. While the prostatic urethra dose was not limited, we found that, by restricting the prescription isodose line to ≥75%, we were able to reduce the prostatic urethra dose to 133% of the prescription dose (27, 28). Target position was verified every 30–60 s during each treatment using paired, orthogonal x-ray images (29).

### Follow-up and statistical analysis

Lower urinary tract symptoms (LUTS) and QOL data were collected for each patient prior to treatment and during routine follow-ups at 1, 3, 6, 9, and 12 months and then bi-annually. LUTS were assessed with the AUA Symptom Score, which ranges from 0 to 35, with higher values representing worsening urinary symptoms (30). QOL data included completion of the Short Form-12 Health Survey (SF-12) (31), the AUA Symptom Index (30), and the Expanded Prostate Cancer Index Composite (EPIC)-26 (32). Dysuria was assessed before and after treatment based on the patient reported response to Question 4b on the EPIC-26 (How big a problem, if any, has pain or burning with urination been for you during the last 4 weeks?). The EPIC summary scores for the dysuria domain range from 0 to 100, with lower values representing worsening dysuria. The responses to this question were grouped into three clinically relevant categories as previously described (33): moderate to big problem (0–40), very small to small problem (41–80), and no problem (81–100).

The EPIC and AUA score minimally important difference (MID) was defined as a change of one-half SD from the baseline (34). Statistical differences in dysuria and AUA scores were assessed using the Student’s *t*-test and chi-square analysis. Univariate and stepwise multivariate analyses were performed to assess dysuria correlation with demographic and treatment variables as well as with other urinary symptoms. QOL data time point patient response numbers are included in Table 3.

## Results

Between 2007 and 2010, 204 patients received SBRT monotherapy for treatment of localized prostate cancer, with a median clinical follow-up of 47 months (range, 10–72 months). Their baseline characteristics are summarized in Table 1. Our patients were ethnically diverse, including 54% Caucasian and 39% African American males. Median age was 69 years (range, 48–91 years). By D’Amico classification, 82 were low-, 105 intermediate-, and 17 high-risk patients. Thirty patients (15%) also received androgen deprivation therapy (ADT). About 88% of the patients were treated with 36.25 Gy in five 7.25 Gy fractions.

**Table 1:** **Patient characteristics**.

		%	*N* = 204
Age (years)	Median 69 (48–91)
	Age ≤ 60	13	27
	60 < Age ≤ 70	45	92
	Age > 70	42	85

Race	White	54	111
	Black	39	79
	Other	7	14

Charlson comorbidity index	CCI = 0	70	137
	CCI = 1	21	42
	CCI ≥ 2	9	18

Median prostate volume (cc)	39 (11.6–138.7)		

BMI	Median 27.5 (15.02–44.96)		

α_1A_ inhibitor usage		18	35

Partner status	Married/partnered	74	151
	Not partnered	26	52

Risk groups (D’Amico)	Low	40	82
	Intermediate	52	105
	High	8	17

ADT		15	30

SBRT dose	36.25 Gy	88	180
	35 Gy	12	24

Baseline QOL demographics are shown in Table 2. The majority of our treatment population reported either mild (50%) or moderate (44%) baseline urinary bother, with a mean AUA score of 8.48 ± 6.12 (range, 0–33). Pre-treatment mean EPIC dysuria assessment revealed that our patient population had baseline minimal dysuria (score 96). Our patient group baseline SF-12 scores were comparable to those of a similarly aged general population (35).

**Table 2:** **Baseline quality of life characteristics**.

Baseline AUA score	*%* Patients (*n* = 204)		
0–7 (Mild)	50%		
8–19 (Moderate)	44%		
≥20 (Severe)	6%		

**Baseline SF-12 score**	**Mean (range)**	**SD**	

PCS	50 (15.6–64.4)	8.76	
MCS	57 (27.2–69.5)	6.71	

**Baseline EPIC-26 dysuria (4b)**	**Mean (range)**	**SD**	**MID**

	96 (25–100)	11.7	5.9

**Figure 1:**
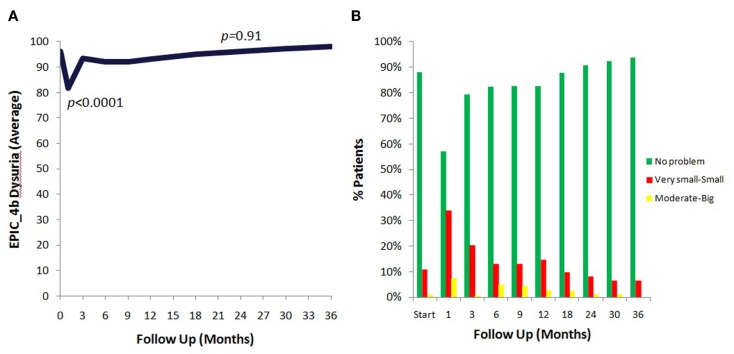
**EPIC urinary dysuria quality of life changes after SBRT**. **(A)** Epic 4b scores before and after SBRT treatment. **(B)** Patients were stratified to three groups: moderate–big (0–40), very small–small (41–80), and no problem (81–100).

The prevalence of patient reported dysuria prior to and after treatment is shown in Table 3. At baseline, 12% of our cohort reported some level of dysuria, with 1% of those patients feeling it was a moderate to big problem. Levels of patient reported dysuria increased significantly following treatment (Figure 1A; Table 3), with 43% of patients reporting dysuria at 1 month (*p* < 0.0001), and 9% of patients reporting dysuria as being a moderate to big problem (Figure 1B; Table 3). There were two distinct peaks of moderate to severe dysuria bother at 1 month and at 6–12 months (Figures 1A,B), with 9% of patients reporting a late transient dysuria flare that peaked at 6–9 months. While a low level of dysuria was seen through the first year of follow-up, our 18-month dysuria scores were virtually identical to the baseline values (Figure 1A; Table 3).

**Table 3:** **Urinary dysuria bother following SBRT for prostate cancer**.

	Start	1	3	6	9	12	18	24	30	36
No problem (%)	88	57	79	82	83	83	88	91	93	94
Very small-small (%)	11	34	20	13	13	15	10	8	6	6
Moderate-big (%)	1	9	1	5	4	2	2	1	1	0
Patient response (*N*)	203	200	198	186	185	178	165	175	171	157

**Figure 2:**
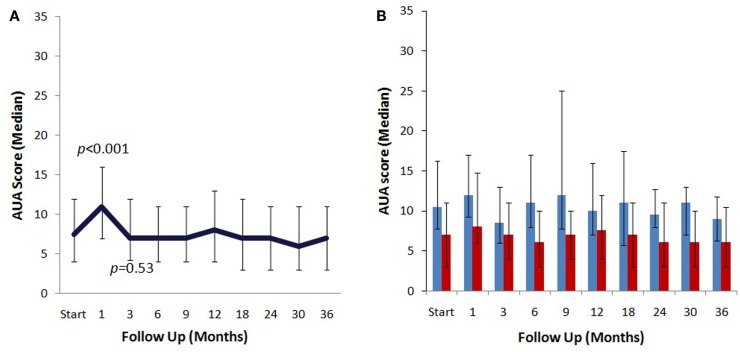
**AUA changes after SBRT**. **(A)** AUA values for the entire cohort prior to after treatment with SBRT. **(B)** AUA values in patients with (blue) and without (red) reported dysuria. AUA scores range from 0 to 35, with higher values representing worsening urinary symptoms.

The median baseline AUA score of 7.5 significantly increased to 11 at 1 month (*p* < 0.0001) and returned to 7 at 3 months (*p* = 0.54) (Figure 2A). Another small peak was seen at 12 months, where the median AUA increased from 7 to 8 (*p* = 0.36). Figure 2B and Table 4 show assessments of AUA scores in patients with and without reported dysuria, revealing that dysuria reporting patients had significantly higher AUA scores at all time points. In addition, the second AUA peak appeared to occur at 9 months in those patients reporting dysuria, consistent with the second late transient dysuria flare revealed in the EPIC questionnaire data (Figure 1).

**Table 4:** **Average AUA after SBRT in patients with and without dysuria**.

	Start	1	3	6	9	12	18	24	30	36
AUA without dysuria	8.07	10.13	7.81	7.04	7.84	8.71	7.7	7.73	7.57	7.87
AUA with dysuria	11.92	13.65	10.02	12.67	14.75	11.87	12.7	11.14	11.08	10.5
*p*-Value	0.011	<0.0001	0.033	<0.0001	0.0003	0.02	0.02	0.03	0.04	0.26

Of the clinical and treatment variables in Table 5, the only predictors of dysuria at 1 month on multivariate analysis were the dose of radiation and the AUA score at 1 month. Initial AUA score did not predict for the development of dysuria. Patients who received 36.25 Gy were significantly more likely to report dysuria than those that received 35 Gy. Table 6 shows the results of a stepwise multivariate analysis comparing the patient reported symptom of dysuria to the individual questions in the AUA questionnaire. While the AUA symptoms of incomplete emptying, frequency, urgency, and straining were significant on univariate analysis, only the AUA symptoms of frequency and strain significantly correlated with dysuria on stepwise multivariate analysis (Table 6).

**Table 5:** **Univariate and multivariate analysis**.

Factors	*p*-Values	OR	95% CI
Age >70	0.213	0.68	0.37
Race	0.07	0.58	0.32
D’Amico’s risk groups	0.724	1.21	0.42
Prostate volume	0.486	0.99	0.98
Charlson comorbidity index	0.301	1.67	0.63
BMI	0.406	1.30	0.70
Dose	0.030^a,b^	3.99	1.15
Initial AUA	0.971	0.99	0.95
AUA at 1 month	0.001^a,b^	1.08	1.03
Initial α_1A_ antagonist usage	0.581	0.80	0.36
α_1A_ antagonist usage at 1 month	0.152	1.54	0.85

^a^Significant on univarariate analysis.

^b^Significant on multivariate analysis.

**Table 6:** **Univariate and stepwise multivariate analysis for AUA correlation**.

AUA questions	*p*-Values	OR	95% CI
Incomplete emptying	0.004^a^	3.42	1.50
Frequency	0.012^a,b^	13.52	1.78
Intermittency	0.774	1.09	0.59
Urgency	0.012^a^	3.05	1.27
Weak stream	0.642	1.18	0.58
Straining	0.0007^a,b^	2.85	1.56
Nocturia	0.343	2.15	0.44

^a^Significant on univarariate analysis.

^b^Significant on multivariate analysis.

## Discussion

Dysuria is a well-known side effect after external beam radiation and brachytherapy (4); however, the incidence and severity of dysuria have not been sufficiently reported after SBRT. SBRT prostate treatment is typically delivered in four to five large radiation fractions. Treatment safety is achieved via intra-fraction image guidance, which allows reduction of the CTV–PTV margin. A growing body of literature has shown SBRT to be safe and efficacious, with multiple single institutional studies (22, 36, 37) and a multi-institutional Phase I study (24) reporting high rates of biochemical control and low rates of grade 3 and higher toxicities with SBRT. Recently, a grouped series of over 1000 patients treated with 4–5 fraction SBRT reported a 5-year biochemical disease-free survival of 93% in all patients and 99% for the low-risk patients with favorable prognosis (38). Indeed, SBRT treatment utilization is increasing, with more patients preferring the convenience of hypofractionated radiation schedules (39).

While differences in patient reported dysuria may be attributable to variability in measurement metrics, including time points interrogated, questionnaire phrasing, and severity levels reported, dysuria following SBRT was comparable to what has been reported following EBRT and brachytherapy (4). As previously described by McBride et al. (24), our mean AUA scores returned to baseline by 3 months post-treatment. However, a minority of patients reported a clinically meaningful urinary symptom flare occurring greater than 6 months after completion of treatment. The peak of the AUA urinary symptom flare did correlate with the same time point as the small secondary increase in dysuria. Changes in AUA were significantly predictive of patient reported dysuria (Table 5), with the AUA measured symptoms of frequency and straining correlating most closely to dysuria on stepwise multivariate analysis (Table 6).

Dose also correlated with report of dysuria (Table 5). In our opinion, dysuria may be exacerbated by the dose to the prostatic urethra and bladder neck in our relatively inhomogeneous plans, so we have modified our institutional protocol to limit dose to these critical structures. Specifically, we now restrict the maximum prostatic urethra dose to 110% of the prescription dose and prescribe to the ≥80% isodose line of the PTV. In addition, we have decreased the bladder neck dose by reducing the anterior/superior PTV expansion to 3 mm. From our clinical experience, such modifications have reduced the incidence and severity of the late urinary symptom flare and patient reported dysuria without increasing the risk of biochemical failures (27).

Patients in our series generally reported a poor baseline urinary function and high alpha antagonist utilization prior to treatment, which is common in the older populations of most radiation therapy series (40–42). While initial alpha antagonist use did not predict for or against dysuria, other studies have shown that prophylactic tamsulosin use statistically lowered the dysuria severity score (9). To maximize patient comfort, it is now currently our institutional policy to initiate alpha antagonists prior to treatment.

Limitations in our study include our high rate of alpha-antagonist utilization (43) and the poor correlation between alpha antagonist utilization and dysuria. Indeed, as we often initiate alpha antagonists to maximize patient comfort, we may have masked the true incidence of SBRT patient reported dysuria (14) and may have given alpha antagonists to many patients with only mild dysuria. In addition, dysuria was commonly transient and the associated bother may have been missed due to the timing of questionnaire administration.

## Conclusion

The rate and severity of dysuria following SBRT are comparable to patients treated with other radiation modalities. Dysuria significantly correlates with dose of SBRT and AUA score, specifically the symptoms of frequency and straining. Our institution practice now includes prophylactic initiation or increase in alpha antagonists to symptomatically manage dysuria. These research findings add to a growing body of literature showing no significant detriment in quality of life measurements with SBRT treatment of localized prostate cancer.

## Author Contributions

EJ is the lead author, who participated in data collection, data analysis, manuscript drafting, table/figure creation, and manuscript revision. TK aided in statistical analysis. LC aided in the quality of life data collection and maintained the patient database. JK aided in the quality of life data collection and maintained the patient database, aided in data collection, and participated in initial data interpretation. TY aided in the quality of life data collection. BC participated in the design and coordination of the study. SS aided in quality of life analysis and manuscript revision. AD is a senior author who aided in drafting the manuscript. JL is a senior author who aided in drafting the manuscript. SC was the principal investigator who initially developed the concept of the study and the design, aided in data collection, drafted and revised the manuscript. All authors read and approved the final manuscript.

## Conflict of Interest Statement

Sean P. Collins and Brian Timothy Collins serve as clinical consultants to Accuray Inc. The authors declare that they have no competing interests.

## Abbreviations

ADT, androgen deprivation therapy; AUA, American Urological Association; BPH, benign prostatic hypertrophy; CT, computed tomography; CTV, clinical target volume; DVH, dose-volume histogram; EBRT, external beam radiation therapy; EPIC, expanded prostate index composite; EQD2, equivalent dose in 2 Gy fractions; GTV, gross target volume; GU, genito-urinary; Gy, gray; IGRT, image-guided radiation therapy; IMRT, intensity modulated radiation therapy; IRB, institutional review board; LUTS, lower urinary tract symptoms; MID, minimally important difference; MRI, magnetic resonance imaging; PTV, planning target volume; QOL, quality of life; SBRT, stereotactic body radiation therapy; SD, standard deviation; SF-12, short form health survey-12-item.
